# Medical loss ratio as a potential regulatory tool in the Israeli healthcare system

**DOI:** 10.1186/s13584-015-0009-8

**Published:** 2015-05-01

**Authors:** Tzahit Simon-Tuval, Tuvia Horev, Giora Kaplan

**Affiliations:** Department of Health Systems Management, Guilford Glazer Faculty of Business & Management, Ben-Gurion University of the Negev, Beer-Sheva, Israel; The Unit of Psychosocial Aspects of Health, The Gertner Institute for Epidemiology and Health Policy Research, Tel-Aviv, Israel

**Keywords:** Medical loss ratio, Voluntary health insurance, Regulation

## Abstract

**Background:**

The growth of the private health insurance sector in Western countries, which is characterized by information deficiencies and limited competition, necessitates the implementation of effective regulatory tools. One measure which is widely used is the medical loss ratio (MLR). Our objective was to analyze how MLR is applied as a regulatory measure in the Israeli voluntary health insurance (VHI) market in order to promote the protection of beneficiaries. The study will examine MLR values and the use of this tool by regulators of VHI in Israel.

**Methods:**

Descriptive analysis using 2005–2012 data from public reports of the Ministry of Health and the Ministry of Finance on VHI plans in three market segments: nonprofit health plans, group (collective) policies offered by commercial insurance companies and individual policies offered by commercial insurance companies.

**Results:**

In 2012, 74% of the Israeli population owned VHI provided by nonprofit health plans and 43% owned VHI offered by for-profit commercial companies. At that time the MLRs of three nonprofit health plans were significantly lower than 80%, mostly in the upper layers of coverage. The MLR in the individual commercial segment was consistently low (38% in 2012). The use of MLR as a regulation tool was, and continues to be, relatively limited in all segments.

**Conclusion:**

The VHI in Israel covers several essential services that are not covered by the statutory benefits package as a result of budget constraints. Thus, due to the high penetration rate of VHI in Israel compared to European countries and the lower levels of MLR, in order to assure the protection of beneficiaries it may be warranted to increase the extent of regulation and adjust it to the nature of the services covered. This may include distinguishing between essential and nonessential coverages and implementation of the most suitable regulatory measures (such as an MLR threshold, limitation of services covered and adjusting the actuarial models to the beneficiaries’ behavior), rather than focusing only on assuring solvency.

## Background

Following the enactment of the National Health Insurance Law (1995). In Israel, all permanent residents have universal coverage providing access to a broad benefits package, including physician consultations, hospitalization, medication and ambulatory care. Health services that are not included in this coverage are financed via voluntary health insurance (VHI) and direct out-of-pocket payments. There are two forms of VHI in Israel. The first is VHI provided by all four health plans^a^ (Clalit Health Services [henceforth Clalit]; Maccabi Healthcare Services [henceforth Maccabi]; Meuhedet and Leumit). The second form is commercial VHI, provided by commercial insurance companies [1]. There is widespread consensus that VHI eases the pressure on the public budget, enables free choice of health insurance and improves efficiency [2], yet most also believe that this market should be regulated in order to assure consumer protection.

The World Health Report [3] encouraged health systems worldwide to be responsive to people’s expectations and preferences. Pursuing this recommendations in the context of VHI means exploration of whether health insurance attributes (i.e. payment modalities, healthcare provider network and management structures) satisfy consumers’ expectations and preferences enough to make it a worthwhile purchase and preferable to other commodities. Theoretically, VHI plans that operate under conditions of perfect competition would inherently strive to satisfy consumers’ preferences; thus, only minimal regulation would be required. However, due to several market failures such as information deficiencies, moral hazard, adverse selection and higher market concentration, more substantial market regulation is warranted.

The medical loss ratio (MLR) is a widely used indicator that is defined as the ratio of total losses incurred (paid and reserved) in medical claims plus adjustment expenses divided by the total premiums earned [4]. Although this measure is calculated differently across countries as well as across different health insurance plans within the same country, generally high ratios can be achieved either through high medical expenditures (numerator) and/or low premiums (denominator). This measure is interpreted by purchasers, insurers and regulators in different manners. In his illuminative article, Robinson [5] has argued that MLR may not be interpreted as a measure of clinical quality or social contribution. He pointed out four potential sources of differences in MLRs: the nature of the vertical structure of the health system, the product diversification, the channel diversification and the geographic scope [5]. Namely, higher MLRs may be observed among HMOs where the insurer and the providers are vertically integrated, since they may attribute less administrative expenses to the insurance plan. In addition, higher values of MLRs may be observed in large health plans or those targeted at large group of employers due to economies of scale and lower marketing costs.

Regulatory approaches to VHI markets vary across countries and on the level of intensity spectrum. At its one end lies a regulatory system that is focused mainly on financial aspects such as enforcement of solvency standards, thus reflecting limited governmental involvement in the market. At the other end of the spectrum lies tighter regulation (material regulation) that adopts stricter requirements to assure consumer protection such as high accessibility to qualitative coverage. The Third Directive of the European Union on Non-Life Insurance in Europe (1994) was adopted by the member states regarding private health insurance markets provided by both commercial and non-commercial insurers. Generally, when addressing health insurance policies that are not substitutive, i.e. do not cover the same benefits as those provided by the statutory health insurance coverage, this directive focuses mainly on financial regulatory tools to promote both the financial stability of insurers and consumer protection. However, when private health insurance is substitutive to the statutory one, tighter regulatory approaches (material regulation) are allowed by the Directive [6]. An example for material regulation can be observed in the Patient Protection and Affordable Care Act Public Law (2010) in the United States, which enforces large‐group health insurance plans (101 employees or more) to keep minimum MLR of 85% of the premium revenue and small‐group (up to 100 employees) health insurance plans to keep a minimum MLR of 80% [7].

In recent years, policy makers in Israel have raised concerns regarding the negative influence of the increase of private health insurance on the publicly financed health sector (e.g. unequal competition on human resources, inefficient duplicity of services, etc.) and recommended that tighter regulation requirements be applied [8-10]. The Israeli setting can provide laboratory conditions to examine the intensity of regulation on the VHI market since it is not subject to exogenous limitations. On the one hand, similarly to most of the European countries, Israel has universal statutory health insurance with relatively generous coverage. In general, its VHI is not a duplication of the statutory coverage or substitutive to it, and its main components are supplementary and complementary to the basic coverage. In addition, similarly to Europe, VHI in Israel is offered both by nonprofit mutualities and by for-profit commercial insures. However, as opposed to the European VHI market, Israel’s regulatory authorities are not obligated to the Third Directive on Non-Life Insurance and thus free to determine the nature and extent of applied regulatory measures, namely, the optimal mix of financial and material regulations. Given the above mentioned calls for more intensive regulation of the VHI market in Israel, one would expect that material regulation will be increased. In this article we examine the intensity of the regulation through one potential regulatory tool – MLR – that combines aspects of consumer protection, including accessibility to quality care, and insurers’ solvency. The purpose of this study is to analyze how MLR is regulated in our unique environment in order to improve the protection of beneficiaries. Specifically, the purpose is to examine MLR values and the degree of its regulation in three market segments of VHI in Israel: the nonprofit health plans, the group (collective) commercial health insurance market and the market of individual commercial health insurance policies. We used 2005–2012 data from public reports of the Ministry of Health and the Ministry of Finance.

### VHI in Israel

Our data refer to VHI for medical expenses (excluding long-term care, compensation for critical illnesses, disabilities, and insurance of non-residents of Israel and for Israelis traveling abroad^b^) of three insurance arrangements: VHI provided by health plans; group (collective) commercial VHI; and individual policies of commercial VHI. Table 1 summarizes the characteristics of these three segments. The VHI provided by the health plans in Israel is subject to a different legal framework compared to the commercial VHI, and while the health plans’ VHIs are regulated by the Ministry of Health which generally implements tight requirements that might be considered as more material regulations, the commercial VHIs are regulated by the Ministry of Finance, which applies less intense requirements and tends to focus more on financial regulation. Healthcare services covered by all three segments may overlap and each VHI may include both complementary coverage (i.e. services exclude from the statutory benefit package and/or reimbursement of statutory charges) and supplementary coverage (greater choice and higher accessibility to services included in the statutory benefit package) [6,11,12]. Although the health plans’ VHIs are provided by nonprofit organizations and the group commercial VHIs are provided by for-profit firms, both provide short-term contracts that can be modified periodically (premium levels and coverage) and both apply community rating as a basis for their premiums. In addition, both segments usually do not require medical underwriting. Although the third segment, i.e. the individual commercial VHIs, are provided by for-profit firms and share the same legislative basis as the group commercial VHI, they are significantly different from the group commercial VHIs due to the fact that they assure lifetime contracts (including guaranteed premiums and coverage) that are adjusted to personal risks (at enrollment) and enrollment is conditional to a medical underwriting process.

**Table 1:** **Characteristics of the voluntary health insurance market in Israel**

**Market segment**	**Health plans**	**Group commercial**	**Individual commercial**
Legal framework	National Health Insurance Law (1994)	Insurance Contract Law (1981) Insurance Business (Control) Law (1981)	Insurance Contract Law (1981) Insurance Business (Control) Law (1981)
Regulatory Authority	Ministry of Health	Ministry of Finance	Ministry of Finance
Medical underwriting is legally possible	No	Yes	Yes
Eligibility	Health plans’ enrollees	Organization (group) members	Whole population
Premium level	Community rating	Community rating	Risk adjusted
Contractual horizon and flexibility	Short-term, periodically modifiable (both premium and coverage)	Short-term, periodically modifiable (both premium and coverage)	Guaranteed lifetime contract, relatively non-modifiable
Tax status	Nonprofit	For-profit	For-profit
Number of firm	4	6	10
CR3^†^	95%^*^	95%^**^	69%^**^
Herfindahl-Hirschman Index^#^	0.35^*^	0.40^**^	0.21^**^
Channel diversification	Single contract to a large group	Single contract to a large group	Different contract to each individual
Vertical structure	Partial ownership of most of providers	Contractual linkage with providers	Contractual linkage with providers

^†^The index measures the fraction of market income (paid premiums) that is attributed to the three largest firms.

^#^The index is calculated as the sum of squared share of all firms operating in the market. The share refers to the ratio of firm’s premiums income in the total income in the market.

^*^Computed by the authors following data from 2013 public report of the Ministry of Health [11].

^**^Source: 2013 public report of the Ministry of Finance [12].

The health plans’ VHIs in Israel are provided by all four health plans. Purchasing VHI from a health plan is conditional to enrollment in the health plan. Clalit, Maccabi and Meuhedet provide both lower and upper layers of coverage, whereas Leumit provides two parallel plans. Purchasing the upper layer of coverage in Clalit, Maccabi and Meuhedet is conditional to purchasing the lower level. Compared to the upper layer of coverage, the lower layer is quite similar (although not identical) across all health plans. Generally, both layers include relatively essential health services that were not included in the statutory coverage due to budget limitation. The lower layer of the coverage in Clalit is called “Mushlam” and the upper one is called “Platinum”. The lower and upper layers of coverage in Maccabi are called “Silver Shield” and “Gold Shield”^c^ respectively. The lower layer of coverage in Meuhedet is called “Adif” and the upper one is called “Meuhedet Si”. The parallel plans in Leumit are called “Silver” and “Gold”. According to the Herfindahl-Hirschman Index (Table 1), the level of concentration in the health plans’ segment is relatively lower than in the group commercial segment, where 6 firms operate (0.35 vs. 0.40), although according to the CR3 index 95% of income in both market segments is attributed to three organizations. A lower level of concentration is observed in the individual commercial segment where 10 firms operate (0.21 vs. 0.35) and 69% of this market segment’s income is attributed to the three largest firms. The vertical structure varies in the VHI market, both between and within segments. While all health plans employ their own physicians, only Clalit and Maccabi own hospitals. On the other hand, the commercial firms (both group and individual) rely primarily on contractual rather than ownership linkage with providers.

Insurers in all segments of the VHI market are obligated to report, to the regulator, data on several financial indicators as well information about the health services covered [11,12]. However, based on current laws and regulatory circulars, neither tight regulation of MLR, such as a minimum threshold or an MLR band, nor imposed sanctions, are observed in this market. In addition, the coordination between the Ministry of Health and the Ministry of Finance regarding the regulations in the VHI market using the MLR is limited; there is no joint standardization of accounting, administration and clinical practices and definitions.

## Results

The number of people enrolled in VHI of health plans in Israel is steadily growing, yet change rates have decreased since 2009 (Table 2). In 2012 74% of the Israeli permanent residents owned at least one policy of VHI provided by the nonprofit health plans. The rates of self-reported ownership of commercial VHI are presented in Table 3. In 2012 43% of the Israeli population reported that they owned commercial VHI. These rates are high compared to OECD countries [13].

**Table 2:** **The number of people enrolled in health plans’ VHI** ^*^ **, Israel, 2005-2012** ^**#**^

***Year***	**2005**	**2006**	**2007**	**2008**	**2009**	**2010**	**2011**	**2012**
n (% of permanent residents)	4.848 (69%)	5.001 (70%)	5.185 (72%)	5.389 (73%)	5.475 (73%)	5.578 (73%)	5.689 (73%)	5.808 (74%)
Change rate		3.2%	3.7%	3.9%	1.6%	1.9%	2.0%	2.1%

^*^Including medical expenses (excluding: long-term care, compensation for critical illnesses, disabilities and insurance of non-residents of Israel and for Israelis traveling abroad) in both lower and upper layers of coverage in Clalit, Maccabi and Meuhedet, and both plans in Leumit (in millions).

^#^Source: [11,14,25-29].

**Table 3:** **The rate of self-reported ownership of commercial VHI, Israel, 2005-2012** ^*^

**Year**	**2005**	**2007**	**2009**	**2012**
% of population	34%	32%	35%	43%

^*^Source: Myers**-**JDC-Brookdale Institute’s survey among adult population (≥22 years old). For this survey VHI included: medical expenses, dental care and compensation for critical illnesses. Presented at The 13^th^ Dead Sea Conference on National Health Policy, December 2012.

The MLR in the VHI provided by nonprofit health plans is calculated as a quotient of actual claims (deducted by copayments) with respect to premiums revenues. The total MLR in all health plans’ VHI was 82% in 2012 [11], yet there is a variance in MLRs across health plans (Table 4). Clalit and Meuhedet, where both layers of coverage actively recruit beneficiaries, demonstrated higher MLR in their lower layer of coverage as compared to the upper layer (87% vs. 72% in Clalit and 96% vs. 45% in Meuhedet). Most of the enrollees in Maccabi and Leumit in 2012 owned the Gold layer of coverage, yet while the MLR demonstrated in Maccabi was 87% in 2012, this measure in Leumit was 69%. In addition, while this measure was relatively steady since 2005 in Clalit and Maccabi, it decreased in Meuhedet and Leumit.

**Table 4:** **Medical loss ratios** ^*^ **of VHI, by health plan, 2005-2012** ^**^

**Health plan (% of the Israeli permanent residents in 2012)**	**VHI (% of the Israeli permanent residents in 2012)** ^**#**^	**2005**	**2006**	**2007**	**2008**	**2009**	**2010**	**2011**	**2012**
Clalit (52.3%)	Mushlam (36.4%)				81%	85%	85%	89%	87%
	Platinum (13.4%)^§^				37%	41%	51%	59%	72%
	**Total**	**82%**	**82%**	**76%**	**75%**	**77%**	**76%**	**80%**	**83%**
Maccabi (25.0%)	Silver Shield (0.5%)				88%	93%	89%	91%	90%
	Gold Shield (21.1%)^§^				88%	95%	92%	95%	87%
	**Total**	**89%**	**89%**	**88%**	**88%**	**94%**	**90%**	**93%**	**87%**
Meuhedet (13.6%)	Adif (9.3%)				81%	81%	80%	89%	96%
	Si (5.4%)^§^				33%	53%	57%	44%	45%
	**Total**	**88%**	**79%**	**75%**	**69%**	**73%**	**73%**	**74%**	**79%**
Leumit (9.1%)	Silver (0.5%)				57%	53%	57%	70%	69%
	Gold (5.7%)				65%	67%	67%	69%	69%
	**Total**	**79%**	**75%**	**71%**	**64%**	**67%**	**67%**	**69%**	**69%**

^*^The MLR is calculated as quotient of actual claims (deducted by copayments) with respect to premiums revenues.

^#^Percentages do not add up to 100% since people may be enrolled in both layers of health plans’ VHI.

^§^Clalit Platinum was launched in 2007; In May 2012 Maccabi’s Silver Shield coverage was closed for new enrollment and united with Maccabi Gold Shield; Meuhedet Si and Leumit Gold were launched in 2004.

^**^Source: [11,14,25-29].

Unlike the calculation method adopted by the health plans, the MLR in the commercial VHI is calculated as a quotient of actual gross claims plus changes in outstanding claims plus indirect expenses for claims management (all included in the numerator) with respect to premium revenues (the denominator). In spite of this difference, these two calculation methods are comparable to some extent since, according to the Ministry of Finance, the components of the changes in outstanding claims and the claims’ management expenses in the numerator are relatively marginal. The MLRs of group and individual commercial VHI in 2012 were 85% and 38%, respectively, and both had increased since 2005 (Table 5).

**Table 5:** **Medical loss ratios** ^*^ **of the commercial VHI, 2005-2012** ^**#**^

**VHI**	**2005**	**2006**	**2007**	**2008**	**2009**	**2010**	**2011**	**2012**
Individual	30%	31%	33%	36%	36%	35%	38%	38%
Group	77%	73%	69%	69%	71%	71%	84%	85%

^*^The MLR is calculated as quotient of actual gross claims plus changes in outstanding claims plus indirect expenses for claims’ management (all included in the numerator) with respect to premiums revenues (the denominator).

^#^Source: [12,16-18,30-33].

The description of the operation, marketing, general and administrative expenses as percentage of premiums’ income is depicted in Table 6 (this data was not available for the commercial VHI). The proportions of these expenses in Meuhedet and Leumit are relatively higher than those in Maccabi and Clalit. In addition, according to the National Health Insurance Law, all VHI provided by health plans must maintain a balanced profit and loss statement. The net profit (or loss) of all health plans is depicted in Table 7. Clalit’s VHI plan demonstrated consistent profits since 2007. Maccabi has demonstrated profits in 2005–2008, had deficits in 2009–2011 and returned to present profit in 2012. Meuhedet has demonstrated consistent profits since 2006 except in 2012 and Leumit has demonstrated consistent profits since 2005 (this data was not available, even in an aggregate level, for the commercial VHI plans).

**Table 6:** **VHIs’ expenses distribution by health plan, 2012** ^*^

**Health plan**	**Clalit**	**Maccabi**	**Meuhedet**	**Leumit**
Health care expenditures (co-payments deducted)	83%	87%	79%	69%
Operation, marketing, general and administrative expenses	14%	13%	21%	27%
Surplus/deficit and financing net expenses	4%	0%	0%	3%

^*^Source: [11].

**Table 7:** **Net profit of VHI by health plan (in millions NIS), 2005-2012** ^*^

**Health plan**	**2005**	**2006**	**2007**	**2008**	**2009**	**2010**	**2011**	**2012**
Clalit	−16	−15	17	30	62	128	97	68
Maccabi	16	18	2	16	−39	−21	−60	6
Meuhedet	−14	4	11	16	11	9	17	0
Leumit	5	16	23	16	9	11	8	9

^*^Source: [11,14,25-29].

## Discussion

Our descriptive analysis reveals high rates of possession of VHI in Israel. In addition, although MLR is not calculated in a uniform method, its values across market segments are comparable to some extent and values lower than 70% (and even lower than 50%) are consistently observed both in policies provided by nonprofit health plans and individual policies provided by commercial for-profit health insurance companies. Finally, the intensity of regulation of MLR is relatively low as reflected by the absence of MLR thresholds and imposed sanctions. The following discussion refers to these insights in light of extant literature.

### Adjusting the regulation intensity to the VHI characteristics

The regulatory approach that is applied in VHI market segments should be associated with the segment role. Namely, if publicly financed statutory universal health insurance with generous coverage exists, it is assumed that VHI plans operate at the margins. In this case, financial regulation that mainly assures solvency may be sufficient to protect the beneficiaries. If on the other hand, no universal coverage exists and most of the health care services are covered via VHI, material regulation should be adopted in addition to the financial one. Examples for this rationale can be found in the US and Europe. The US health care system, which was mostly based on privately financed VHI, enforced material regulation through the Patient Protection and Affordable Care Act by determining, for example, that health insurance plans that do not meet a targeted MLR threshold must provide rebates to their policyholders [7]. As opposed to this kind of material regulatory approach, no threshold is imposed in European countries, where the VHI market provides complementary and/or supplementary coverage to the universal one and allows more material regulative interventions targeted at statutory or substitutive to statutory schemes [6]. The Israeli VHI market resembles the European one, though it covers several essential services that are not covered by the statutory benefits package as in Europe such as dental care for children (older than 12 years old) and the elderly or physiotherapy and child development services that are limited in the statutory coverage. VHI that provides these services might be considered as substitutive to the statutory coverage. Due to the importance of improving accessibility to essential services and given the high penetration rate of VHI in Israel, one would expect that tighter regulatory requirements would be applied. The relatively higher MLRs that were continuously observed in the upper layer of several nonprofit health plans and the group commercial VHI (e.g. 85-90%) may imply that setting high MLR threshold may be feasible in this context. The consistent low levels of MLR observed in several VHIs (45-70%) suggest that this kind of tighter regulatory approach is applied in a limited manner and that regulation is not necessarily adjusted to whether the coverage includes essential services.

Tighter regulation does not necessarily mean a uniform one. It may be warranted to better define the role of each VHI within segments, rather than applying uniform regulatory tools to the whole segment. For example, currently both the lower and the upper layers of coverage that are provided by the nonprofit health plans include essential services. Regulators may monitor the services included in the lower layer and assure that these are mainly essential services that were not included in the statutory coverage due to limited public resources. If this is imposed, then tighter regulatory interventions such as enforcement of MLR threshold could be justified regarding the lower layer, whereas the upper layer may be regulated less tightly (implementing different or no thresholds). In addition, MLR thresholds may be differentiated by the health plan’s size. The next subchapter of the discussion will analyze the primary reasons for lower MLRs. These include: continuous increase in premiums that is justified by continuous broadening of coverage; diseconomies of scale; guaranteed lifetime contracts and market concentration. In addition, it suggests corresponding material regulatory tools.

### Potential sources of low MLRs and suggested intervention tools

Our descriptive analysis reveals that MLRs lower than 80% are observed both in VHI provided by nonprofit health plans and VHI provided by for-profit commercial firms. MLRs observed in the nonprofit health plans consistently varied during the study follow-up period both between and within health plans (between coverage layers). The lower rate (72% in 2012) in the upper layer of coverage in Clalit (Platinum) may be attributed to its relatively recent launch date (2007) and to the fact that the annual enrollment rate is still high, thus relatively high proportion of its enrollees did not complete their waiting period and cannot submit medical claims. This explanation is not applicable to the Leumit (69% in 2012) and Meuhedet (45% in 2012) plans since their launch date was 2004, and their enrollment rates are relatively low. Thus, most of the beneficiaries already completed their waiting periods and are free to submit medical claims [14]. In addition to the influence of the eligibility to submit medical claims that affects the numerator, these low rates may be attributed to an increase in premiums that influences the denominator. Adopting more material regulation could evaluate the services included in both layers of coverage, and if an increase in premiums is justified by the inclusion of additional services to the coverage, this inclusion may be monitored and limited. It is not inevitable that premiums will continuously increase due to additional coverage. If this additional coverage is implemented too frequently and is relatively non-essential, regulators may consider limiting it.

Another reason for the lower MLRs in Leumit and Meuhedet (nonprofit health plans) and the individual commercial VHI is diseconomies of scale which increase marketing, commission and administrative costs for both segments [5,15]. This argument may be reinforced by the evidence of relatively low operational expenses (and higher MLRs) in Maccabi and Clalit as compared to Leumit and Meuhedet, yet material regulation that will enforce disclosure/reporting requirements to promote transparency will enable unbundling the non-medical expenses (administration, marketing, agents’ commissions etc.) and assuring that health plan efficiently allocates its resources toward healthcare utilization of its beneficiaries. In the nonprofit health plans segment, it may be expected that the consistent profits (as observed in Leumit and Meuhedet) may enable an increase in their MLR through broadening coverage or shortening the eligibility waiting period while assuring solvency. As mentioned, data on the distribution of expenses among insurers in the commercial segment (both group and individual) was not available in recent years. However, data for 2005–2008 reveals that on average 12% of premium revenues were distributed for general and administrative expenses, while agents’ commissions comprised an additional 20% of revenues [16-18]. Empirical analysis from the US revealed an inverse relationship between employer group size and its loading fees [19]. Evidence from 2011, the first year under the Affordable Care Act, revealed that US insurers reduced administrative costs nationally, but in the group (collective) markets no reduction in the nonmedical overhead was observed (lower administrative costs were offset by increased profits of a similar amount) [20].

The lower MLRs observed in the individual commercial VHI may predominantly stem from the fact that the individual commercial VHI in Israel are based on guaranteed lifetime contracts, thus are subject to high uncertainty with regard to technological, morbidity and regulation changes. This necessitates caution and reliance on conservative assumptions in the pricing process. As opposed to that, both the health plans and group commercial VHI can periodically change the statute terms (i.e. premiums levels and coverage) as needed. Evidence for this argument was found in the private health insurance market in Europe, where Hungarian health insurance plans that guarantee a lifetime contract demonstrated significantly lower MLR compared to Ireland’s *ceteris paribus* (54% vs. 71% in 2006 [6]). But these low levels of MLR in the individual insurance policies are not inevitable. It should be demonstrated that these lifetime contracts are actually being kept by most of the beneficiaries until the contract ends. If this is not the case and the beneficiaries terminate the contract earlier due to inability to pay (premiums as well as copayments), regulators may request insurers to adjust the actuarial model to the actual behavior of most beneficiaries and this may result in reduction in premiums.

Another reason for relatively lower values of MLR in the individual commercial market relies on the lower bargaining power of the contract owner compared to this power in group commercial VHI (i.e. the employer) in lowering premiums and broadening coverage. However, as described before, the individual commercial VHI segment is less concentrated than the health plans and the group commercial segments as measured by the Herfindahl-Hirschman Index and the CR3 index. Our findings also contradict recent empirical evidence from the US [21,22] of lower MLRs among insurers with monopoly power. This inconsistency may stem from the fact that the analysis in the US was focused on insurers within the individual commercial segment and that more prominent attributes may have influenced health insurance behavior. In addition, although the individual commercial VHI is relatively more competitive than the other two segments, all three segment are operating under imperfect competition with three firms comprising, in terms of income, 69% of the individual commercial market and 95% of the nonprofit health plans and group commercial segment. In the nonprofit health plans segment this problem is aggravated due to the fact that beneficiaries are relatively captive customers, since they are required to be enrolled in the health plan for the statutory coverage in order to purchase its VHI and cannot opt for a VHI of another health plan without changing the entire provision of the statutory health care coverage including primary care physician, pediatrician etc. These conditions necessitate relatively tight material regulation which is targeted at consumer protection.

Although MLR may be used as a regulatory tool to protect consumers, it is well known that it ignores several aspects. These include health gains and the beneficiaries’ experience [5,23]. Both components of MLR – premiums and expenditures – do not exclusively reflect these outcomes [5]. Thus, it is recommended to consider developing an unbiased measure for health gain and for the beneficiaries’ experience both when purchasing health insurance and when submitting claims as part of regulatory intervention. Indeed, in July 2014 the Ministry of Finance in Israel published a service quality index of the commercial VHI plans which focused on the claims handling process. This index refers to the proportions of accepted claims and to the promptness in which they were managed. The World Health Organization (WHO) has defined health systems’ “responsiveness” as comprised of aspects such as: choice, communication, confidentiality, quality of amenities and prompt attention [3,24]. Surveys of these measures may need to be routinely and consistently implemented and reported. Following the estimation of these aspects, there is a need to develop a comprehensive adequate summary measure of VHI’s value. It may be applied by an estimation of the relative weights of different outcomes from the beneficiaries’ point of view.

## Conclusion

The current study analyzed MLR as an indicator that may be adopted by regulators in order to improve the protection of beneficiaries and may reflect the intensity of regulation in the VHI market. VHI in Israel covers several essential services that are not covered by the statutory benefits package due to budget constraints. It is demonstrated that although regulators in Israel are not subject to obligatory restrictions, a less tight regulative approach was adopted in spite of the fact that consistent low levels of MLR are observed. This was found to be the case even in policies that provide several essential services which are not included in the statutory coverage due to public budget constraints. Given the high penetration rate of VHI in Israel, there might be a need to consider adopting tighter material regulation in this market in order to improve the protection of beneficiaries. This regulation may include standardization and transparency of MLR data, adjustments to the nature of the services covered (i.e. through distinction between essential and less essential coverage), imposing MLR thresholds, limitation of services covered and adjusting the actuarial models to the beneficiaries’ behavior. The ability to apply these regulations depends on the existence of transparent and reliable databases and rigorous standardization requirements regarding accounting, administration and clinical practices and definitions.

### Endnotes

^a^These health plans function as insurers as well as providers. In other countries they may be referred to as HMOs (health maintenance organizations), MCOs (managed care organizations) “sick funds” [1] or mutualities [6].

^b^Dental care is excluded from the analysis of policies provided by commercial insurance companies.

^c^In May 2012 Maccabi’s “Silver Shield” coverage was closed for new enrollment and united with Maccabi “Gold Shield”. In February 2013 Maccabi launched an upper coverage, “Maccabi Sheli”. Thus, the lower layer of coverage to date is “Gold Shield” and the upper one is “Maccabi Sheli”.
